# The Mental Health of Adult Irregular Migrants to Europe: A Systematic Review

**DOI:** 10.1007/s10903-022-01379-9

**Published:** 2022-07-15

**Authors:** Fiona Martin, S. P. Sashidharan

**Affiliations:** College of Medical, Veterinary and Life Sciences, University of Glasgow, Glasgow, G12 8QQ Scotland, UK

**Keywords:** Immigration, “undocumented migrants”, “mental health”, “mental disorders”

## Abstract

The aim of this systematic review is to summarise the existing evidence on the mental health outcomes of adult irregular immigrants (IMs) to Europe. Database (MEDLINE, EMBASE, CINAHL, PsychINFO) searches were conducted according to PRISMA. The risk of bias was assessed using the Appraisal tool for Cross-Sectional Studies. The database searches yielded 2982 results. Eight cross-sectional studies from Western Europe were included, with 1201 participants. The prevalence of mental disorders varied between studies: depression from 8 to 86%; anxiety from 3.1 to 81%; and post-traumatic stress disorder (PTSD) from 3.4 to 57.6%. The studies had methodological flaws; in particular a risk of unrepresentative samples. There was methodological heterogeneity, therefore pooling of data, and direct comparisons were not possible. The majority of studies found higher rates of depression, anxiety and PTSD than previous estimates for the general population, and higher rates of depression and anxiety than previous estimates for other migrant groups.

**Supplementary Information:**

The online version contains supplementary material available at 10.1007/s10903-022-01379-9.

## Introduction

The number of international migrants is rising. The United Nations (UN) estimated that there were 281 million in 2020, accounting for 3.6% of the global population, increased from 173 million in 2000. Europe was the region which hosted the largest number of international migrants in 2020, hosting 87 million [1]. The International Organisation for Migration (IOM) has previously estimated that 10–15% of international migrants are irregular migrants (IMs) [2]. The European Commission defines irregular migration as “movement of persons to a new place of residence or transit that takes place outside the regulatory norms of the sending, transit and receiving countries” [3]. There has been a recent surge in irregular migration to Europe; over 1 million migrants arrived by sea in 2015 [4]. This journey is deadly, with more than 2000 migrant deaths in the Mediterranean in 2018 [5].

People migrate for a variety of reasons. Some do so by choice, in order to be reunited with family, or to pursue academic or economic opportunities. However, an increasing number are doing so out of necessity, in order to escape human rights violations, persecution or conflict, or the effects of natural disasters or climate change [6]. These individuals may be unable to return to their country of origin until the situation which they have fled has resolved, and deportation could be potentially life-threatening [7].

Previous research has found that some migrant groups may experience higher prevalence rates of certain mental disorders. A rigorous meta-analysis by Blackmore et al. [8] found that the rates of depression in asylum-seekers and refugees was 31.5%, and of post-traumatic stress disorder (PTSD) was 31.46%, which are higher than estimates for the general population globally, and that these higher rates persisted for many years. Conversely, it found that the rates of anxiety and psychosis were lower. A systematic review by the WHO [7] found that the rates of mood, psychotic and substance use disorders in asylum-seekers, refugees and IMs were similar to those in host populations. An exception was PTSD, for which they found higher rates in migrants. They only identified a limited number of studies which exclusively investigated IMs, and in those that did, the rates of PTSD were similar to host populations. They also found that rates of depression in migrants who had been re-settled for more than 5 years were higher than in host populations, and associated with socioeconomic factors. There was significant variation in the rates between studies; this may represent true differences, or study heterogeneity.

A systematic review by Garcini et al.[9] found that undocumented migrants to the United States of America experienced multiple stressors throughout the migration process, and that psychological distress was common, however, there was limited data on the prevalence of specific mental disorders. The included studies had significant methodological flaws, including varying definitions of IMs, and overreliance on convenience sampling. No previous systematic reviews on the mental health outcomes of adult IMs to Europe were identified.

Migrants are exposed to risk factors for mental health difficulties throughout the migration process [7]. A study by Chen et al. [10] investigating humanitarian migrants to Australia found that they had experienced a mean number of 2.1 traumatic events pre-migration. Post-migration the proportion having experienced: poor social integration, economic difficulties, worrying about friends and family overseas and loneliness, was 64%, 59%, 49% and 18% respectively. Adverse experiences both pre- and post-migration were associated with serious mental illness and PTSD.

A systematic review by Satinsky et al. [11] found underutilisation of mental health services by asylum-seekers and refugees to the European Union (EU). It identified barriers to accessing care including: lack of awareness, help-seeking behaviours, communication difficulties and stigma towards, and by providers. A study which interviewed experts in delivering mental health care to IMs in Europe by Straßmayr et al. [12] identified additional barriers specific to IMs including: lack of legal entitlement to health care in many countries, lack of awareness of such entitlements in others, and fear of deportation.

### Aim

The objective of this systematic review was to summarise the existing evidence on the mental health outcomes of adult IMs to Europe. Mental health outcomes were defined as: the nature and prevalence of mental health difficulties, whilst mental health difficulties were defined as: psychological symptoms, diagnosis with a mental disorder, or poor overall mental health.

## Method

### Search Strategy and Selection Criteria

Databases (MEDLINE, EMBASE, CINAHL and PsychINFO) were searched using keywords and subject headings for IMs, terms related to ‘mental disorder’, and specific psychological symptoms and mental disorders, according to the Preferred Reporting Items for Systematic Reviews and Meta-Analyses (PRISMA) statement [13], between 7th November and 6th December 2020. The full search strategy is included in the review protocol in Appendix 1. The date limits were 1st January 1990 to the time of the searches. This start date was chosen because there was an increase in migration in Europe around this time [14].

Studies were included if: (1) the sample included adult (≥ 18 years old) IMs to Europe, and their data were separated from other participants, including detained migrants, (2) they reported the nature and prevalence of mental health difficulties in the IM participants. Irregular migration was defined from the perspective of host countries as “entry, stay or work in a country without the necessary authorisation or documents required under immigration regulations” (3). Studies were excluded on the following criteria:

1. Samples recruited through mental health services
2. Studies investigating substance use, but not other mental health outcomes
3. Intervention studies
4. Studies with only qualitative data
5. Unable to access full-text or English language versions
6. Non-peer reviewed literature

Where a single data set was reported in multiple articles, only the article which best met the selection criteria was included. The full selection criteria are included in the review protocol in Appendix 1.

One reviewer (FM) assessed the titles, abstracts and full-texts against the selection criteria, and removed duplicates. A second reviewer (SS) independently assessed 5% of the abstracts against the selection criteria; there was complete agreement between reviewers.

### Data Analysis

One researcher (FM) extracted data from the studies relating to: study and participant characteristics, methods of measurement and mental health outcomes. Due to methodological heterogeneity, no pooling of data relating to mental health outcomes was possible.

### Risk of Bias Assessment

One reviewer (FM) used the Appraisal tool for Cross-Sectional Studies (AXIS) to assess the risk of bias. All of the included studies had a cross-sectional design, and AXIS is the only formal tool for the critical appraisal of this study design. Studies were awarded one point per question when high quality methodology was present; the maximum overall score was 20 points. In those studies in which there were no non-responders, questions relating to non-responders were answered “not applicable”, and awarded one point [15]. There is no grading system for AXIS; the following grading system was developed for this review: high quality for > 80% score, moderate for 50–79% and low for < 50%.

## Results

### Search Process

**Fig. 1:**
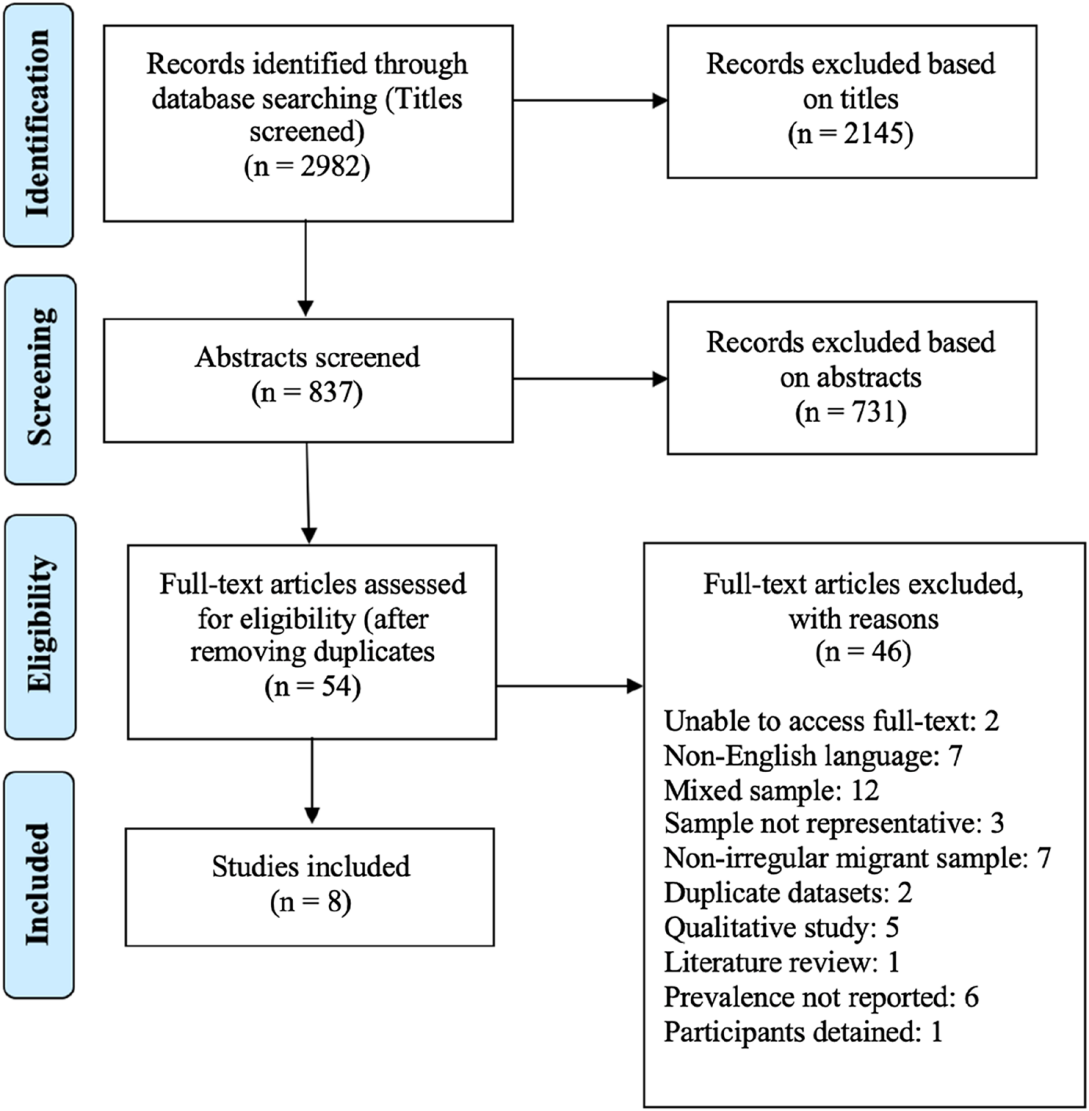
PRISMA flow diagram of the search process

The database searches yielded 2982 results, of which eight studies met the selection criteria. The search process is outlined in the PRISMA diagram in Fig. 1. Studies were excluded for various reasons; seven were excluded because an English language version was unable to be obtained (these articles were written in Italian, German, Spanish and Dutch).

### Study Characteristics

The characteristics of the included studies are presented in Table 1. All studies were conducted by academic institutions in Western Europe, between 2006 and 2020, with the quantitative component of all being of a cross-sectional design. The majority of studies recruited participants by convenience sampling [16–21]. The terms used to refer to IMs varied between studies, some used “undocumented migrants” [18–20, 22, 23], whereas others used terms relating to the illegality of their status [16, 17]. The definition of IMs also varied; in Sousa et al.’s study [23], IMs were defined as not having permission to work in the host country, whereas others used various definitions relating to not having permission to reside [16–20], and Schoevers et al.’s study [22] did not provide a definition. The sample sizes varied from 21 [17] to 438 [23], and the total number of IM participants included in this review was 1201. Some study samples included other migrant groups and non-migrants for comparison.

**Table 1:** Characteristics of included studies

Study	Host country	Study design	Sampling method	Definition of irregular migrant	Sample size (irregular migrants)
Naimo et al. [16]	Italy	Mixed methods (quantitative component was cross-sectional)	Convenience	Migrants who had “entered Italy illegally and under traumatic circumstances”	39
Schoevers et al. [22]	Netherlands	Cross-sectional	Purposive	Not specified	100
Sousa et al. [23]	Spain	Cross-sectional	Quota	No permission to work in Spain	438
Heeren et al. [17]	Switzerland	Cross-sectional	Convenience	Migrants who had entered Switzerland without visas and were living in illegality at the time of the study	21
Teunissen et al. [18]	Netherlands	Cross-sectional	Convenience	Visa ‘overstayers’, rejected asylum-seekers and individuals who had entered the country illegally	325
Myhrvold and Smastuen [19]	Norway	Mixed methods (quantitative component was cross-sectional)	Convenience	Migrants without a residence permit authorising them to regularly stay in the country of destination	90
Andersson et al. [20]	Sweden	Cross-sectional	Convenience	Having applied for asylum and a residence permit but the application had been rejected and the decision gained legal force; persons from outside the European Union having overstayed in Sweden after their visa had expired; or persons having moved to Sweden without applying for a visa	88
Angeletti et al. [21]	Italy	Cross-sectional	Convenience	Migrants rescued in the Mediterranean Sea after attempting to cross by boat from Libya	100

### Risk of Bias

One study was graded high quality [18], six moderate [16, 17, 19, 20, 22, 23], and one low [21] on AXIS. None were awarded points for justifying the sample size, the representativeness of the sampling frame, or the likelihood of the selection process to select a representative sample, and none provided information on non-responders, or took measures to address them.

### Participant Characteristics

43.2% of participants were female. Other participant characteristics could not be pooled due to heterogeneity in how they were presented. There were high levels of trauma exposure [16, 17, 19, 21, 22] in those studies which reported on it.

### Measurement of Mental Health Outcomes

Information relating to the measurement of mental health outcomes is presented in Table 2. Six studies, with 663 participants, measured the prevalence of depression [16–20, 22], five studies, with 624 participants, measured anxiety [17–20, 22], five studies, with 573 participants, measured PTSD [16–18, 20, 22], and three studies measured overall mental health [18, 19, 23].

**Table 2:** Measurement of mental health outcomes in included studies

Study	Method of measurement	Role of assessors	Questionnaire/ interview in participant’s native language
Naimo et al. [16]	SCID for DSM-IV	Not specified	Mixture *Interview in Italian, but in some cases an Albanian interpreter was necessary*
Schoevers et al. [22]	Self-reporting of health problems (spontaneously, then with a list of health problems)	GP	Mixture *Interview in the Dutch or English. For women with inadequate Dutch or English language abilities to participate in the assessment, interpretation was offered (mixture of professional and relatives)*
Sousa et al. [23]	GHQ-12	Professional interviewers	No *The inclusion criteria included adequate Spanish language abilities to participate in the assessment*
Heeren et al. [17]	HSCL-25; HTQ; PDS	Self-administered	Yes
Teunissen et al. [18]	Review of general practice records 2010–2011	Researchers	N/A
Myhrvold and Smastuen [19]	HSCL-25	Self-administered	Mixture *One third filled out the questionnaires in English or Norwegian. The largest language groups at the Health Centre, in addition to Norwegian and English, were selected in advance: Pashto, Mongolian, Farsi, Amharic and Somali*
Andersson et al. (20)	BDI-II; BAI; PCL-5	Trained field workers	Yes
Angeletti et al. [21]	RHS-15	Psychologists	Yes

*SCID DSM-IV* Structured Clinical Questionnaire for DSM-IV, *GHQ-12* General Health Questionnaire-12, *HSCL-25* Hopkins Symptom Checklist-25, *HTQ* Harvard Trauma Questionnaire, *PDS* Posttraumatic Diagnostic Scale, *HSCL-25* Hopkins Symptom Checklist-25, *BDI-II* Beck's Depression Inventory, *BAI* Beck’s Anxiety Inventory, *PCL-5* Post-traumatic stress disorder Checklist, *RHS-15* Refugee Health Screener-15, *GP* General practitioner

The studies used different methods to measure mental health outcomes. Teunissen et al.’s study [18] reviewed general practice records. All other studies used self-report [16, 17, 19–23]. Schoevers et al.’s study [22] used self-reporting of health problems spontaneously, and then with a standard list of common health problems and concise list of chronic diseases; these lists are not known to have been validated. All measures used in the other studies have been validated in multiple populations globally [16, 17, 19–21, 23]. The role of assessors varied between studies, and in two studies assessments were self-administered [17, 19]. Sousa et al.’s study [23]conducted assessments in the host country’s national language (Spanish), three others conducted assessments in participants’ native languages only when necessary [16, 19, 22], and the remainder conducted all assessments in participant’s native languages [17, 20, 21].

### Mental Health Outcomes

**Fig. 2:**
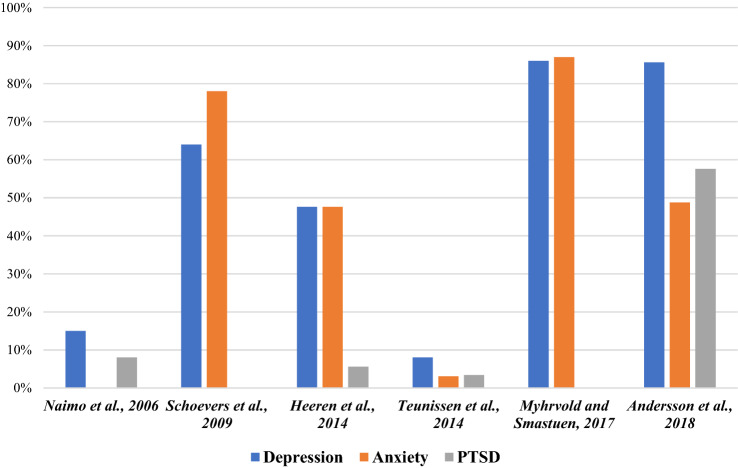
Prevalence rates of mental disorders for irregular migrant participants in included studies

The prevalence rates of depression, anxiety and PTSD are presented in Fig. 2 and Table 3. It should be noted that due to the heterogeneity of the methods of measuring mental health outcomes, direct comparisons are not possible.

**Table 3:** Prevalence rates of mental disorders for irregular migrant participants in included studies

Study	Prevalence of depression	Prevalence of anxiety	Prevalence of PTSD
Naimo et al. [16]	15%	-	8% (partial PTSD 25%)
Schoevers et al. [22]	9% spontaneously reported having experienced depressed mood; 64% reported it when asked specifically	7% spontaneously reported having experienced anxiety; 78% reported it when asked specifically	–
Sousa et al. [23]	–	–	–
Heeren et al. [17]	47.6%	47.6%	Probable PTSD 5.6%
Teunissen et al. [18]	8%	3.1%	3.4%
Myhrvold and Smastuen [19]	86%	87%	–
Andersson et al. [20]	85.6% (mild 14.5%; moderate 13.2%; severe 57.9%)	48.8% (mild-moderate 19.3%; moderate-severe 29.5%)	57.6%
Angeletti et al. [21]	–	–	–

There was significant variation in the prevalence of depression, anxiety and PTSD between studies. Depression ranged from 8% having a diagnosis recorded in their general practice notes between 2010 and 2011 [18], to 86% having a DSM-IV diagnosis on HSCL-25 [19]]. Anxiety ranged from 3.1% having a diagnosis recorded [18], to 81% having clinical levels on HSCL-25 [19]. And PTSD ranged from 3.4% having a diagnosis recorded [18], to 57.6% fulfilling the criteria on PCL [20]. In Schoevers et al.’s study [22] there were significant differences in the rates of mental health difficulties reported spontaneously, compared with when asked specifically. 9% reported experiencing depressed mood spontaneously, and 64% when asked specifically. And 7% reported experiencing anxiety spontaneously, and 78% when asked specifically.

Of those studies which investigated overall mental health, Sousa et al.’s study [23] found that 51.3% of male participants, and 50.6% of females, had poor mental health on GHQ-12. In Myhrvold and Smastuen’s study [19] 87% had a score on HSCL-25, indicating an experience of emotional stress in need of additional diagnostic evaluation and mental health care. In Angeletti et al.’s study [21] 100% of participants had a score on RH-15, and/or Distress Thermometer indicating symptoms of common mental disorders requiring further assessment (Cronbach ‘s *α* 0.95).

Some studies compared the mental health of IMs with that of other migrant groups and non-migrants. Heeren et al.’s study [17] found that whilst IMs had higher rates of depression and anxiety than labour migrants (*p* < 0.05; *p* < 0.01) and residents (*p* < 0.01), they had lower rates of depression and PTSD than asylum-seekers (*p* < 0.001). Teunissen et al. [18] found that IMs contacted their GP less often than documented migrants (3.1 times per year versus 4.9), and that 20.6% of IMs had at least one psychological (P) International Classification of Primary Care code documented in their general practice records between 2010 and 2011, compared with 44% of documented migrants (*p* = 0.00).

Other studies investigated factors associated with mental health outcomes. Naimo et al.’s study[16] found that females experienced major depressive disorder more frequently (*p* = 0.01). Andersson et al.’s study [20] found that those aged 40 years or more had higher depression scores (*p* < 0.05). Additionally, it found that insecure housing post-migration was associated with both depression and anxiety (*p* < 0.05). Myhrvold and Smastuen’s study ([19]found that migrating due to conflict or persecution (*p* < 0.01), having financial dependents (*p* < 0.04), and having experienced homelessness (*p* < 0.04), hunger (*p* < 0.01) and harassment (*p* < 0.03) were associated with higher levels of psychological distress, whilst having a higher level of education was associated with a reduction (*p* < 0.01).

## Discussion

### Strengths and Limitations

This is thought to be the first systematic review investigating the mental health outcomes of adult IMs to Europe. It included a comprehensive search and selection process and was conducted using a systematic approach. However, it is recognised that it had limitations. It was conducted by a single researcher (FM); additional researchers may have enhanced the reliability, in particular of study selection, data extraction and critical appraisal. Specifically, the involvement of additional reviewers in study selection may have increased the number of relevant studies included [24]. However, in order to assess the reliability of the screening process, a second reviewer (SS) screened 5% of the abstracts; there was complete agreement between reviewers. Studies were excluded on the basis of full-text or English language versions being unable to be accessed; this may have led to the exclusion of potentially relevant studies.

There is a lack of critical appraisal tools for cross-sectional studies; AXIS was used as it is the only formal tool. Moskalewicz and Oremus’s study (2020) evaluating AXIS found poor inter-rater reliability, and further evaluation was recommended. Therefore, the reliability of the critical appraisal of studies in this review is unknown. Only one study was graded high quality on AXIS, and a risk of bias was identified across all studies, in particular, a risk of unrepresentative samples [18].

There is no universally agreed definition of an IM; this was reflected in the studies in this review, which had varying definitions. The authors of Sousa et al.’s study [23] explained that they adopted their definition relating to not having permission to work in the host country because for the majority of non-EU migrants, permission to work requires permission to reside, and they thought that this definition would increase participation. However, they acknowledged that the degree of overlap between undocumented work, and undocumented resident populations is unknown. This variation in definitions will have meant that the target population differed between studies.

There are challenges to recruiting IMs to research. The population is largely hidden, and potential participants may have concerns that participation could bring them to the attention of authorities, and risk deportation. Consequently, research in this field relies heavily on non-probability sampling; this was used by the majority of the studies in this review, leading to a risk of selection bias [16–22]. Additionally, three of the studies used organisations which support IMs to recruit participants [16, 20, 22], whilst Myhrvold and Smastuen’s study [19]used a health centre. Participants engaging with support organisations and health services may be more likely to have mental health difficulties, which could lead to an overestimation of prevalence. A systematic review by the WHO [7] found that studies that used convenience sampling found higher prevalence rates of mental disorders than those with more representative samples.

Only eight studies were identified which met the selection criteria. The sample sizes of these were relatively small, as was the total number of IM participants included in this review; this limits the external validity of the results.

There was clinical, methodological and statistical heterogeneity between studies. Differences included: mental health outcomes measured, and the methods by which this was done, role of assessors, whether or not assessments were conducted in participant’s native language, and statistical analysis. This heterogeneity meant that pooling of data on mental health outcomes, and direct comparisons between studies were not possible. Most of the measures used have been validated in multiple populations globally, however, none have been validated specifically in the IM population; therefore, the validity of the results in this population is unknown. Most studies used self-report, [16, 17, 19–23], leading to a risk of self-report bias. Four studies conducted assessments partially, or fully, in languages not native to participants [16, 19, 22, 23]; this will have adversely affected the reliability of the results. Blackmore et al.’s meta-analysis ([8] found that the prevalence rates of depression and PTSD in asylum-seekers and refugees were higher in those studies that used interpreters.

## Conclusions

Eight studies were identified which met the selection criteria for this systematic review, which indicates the lack of research on the mental health outcomes in adult IMs to Europe. Knowledge gaps were identified. The studies focused on the prevalence of depression, anxiety and PTSD, whilst largely neglecting other potentially relevant mental health outcomes, such as psychosis. And they only studied IMs to Western Europe. Therefore, the prevalence of other mental health outcomes in IMs, and the outcomes for IMs to elsewhere in Europe remain unknown.

The majority of studies found higher prevalence rates of depression, anxiety and PTSD than previous estimates for the general population globally [16, 17, 19, 20, 22]. According to data from the WHO World Mental Health Surveys, the global lifetime prevalence of depression is 12%, anxiety disorders 11% and PTSD 3.9% [25, 26]. The exception was Teunissen et al.’s [18] study, which found lower rates for all these disorders. Other studies found IMs had poor overall mental health [19, 21, 23].

Some studies compared different migrant groups. Teunissen et al.’s [18] study found that IMs contacted their GP less often than undocumented migrants. This suggests that greater barriers exist to IMs contacting their GPs. Comparisons in terms of mental health outcomes were mixed. The majority of those studies which investigated depression and anxiety found higher rates than a previous meta-analysis by Blackmore et al. [8] of asylum-seekers and refugees [17, 19, 20, 22], whilst, the majority found lower rates of PTSD [16–18].

There was significant variation in the prevalence of different mental health outcomes. This variability may represent true differences between different IM populations, in different contexts, however, it may also reflect heterogeneity in study methodology and quality. A systematic review by the WHO [7] found that studies of higher methodological quality found lower rates of mental disorders. This is in keeping with this review; Teunissen et al.’s study [18]was the only one graded high quality on AXIS, and found the lowest rates of depression, anxiety and PTSD, whereas Angeletti et al.’s study [21] was the only one graded low quality, and found that 100% of participants had symptoms of common mental disorders requiring further assessment. Another possible explanation for the low rates in Teunissen et al.’s study [18] is its unique method of measuring outcomes: review of general practice records. Contributing factors to these rates may include: barriers to IMs accessing general practice regarding mental health difficulties, language barriers, and the cultural competence of GPs in assessing the mental health of patients from other cultures. The presence of barriers to accessing general practice is supported by the finding that IMs contacted their GP less than documented migrants.

Some studies investigated factors associated with mental health outcomes. Two studies found significant associations between insecure housing post-migration and worse mental health outcomes, however, due to the cross-sectional design this cannot be used to infer causality [19, 20].

In Schoevers et al.’s study [22] there were significant differences in rates of mental health difficulties reported spontaneously, compared with when asked specifically. This suggests that IMs may not report mental health difficulties to healthcare professionals unless specifically asked about them. Contributing factors may include: a lack of awareness of mental health difficulties, and stigma associated with experiencing them, among the IM population. Similarly, this is a possible explanation for the relatively low rates of mental disorders recorded in general practice records in Teunissen et al.’s study [18].

## Recommendations

1. Only a limited number of studies on the mental health outcomes of adult IMs to Europe were identified, and the majority focused on the prevalence of depression, anxiety and PTSD in IMs to Western Europe. Therefore, the prevalence of other mental health outcomes, and the outcomes for IMs to elsewhere in Europe, remain unknown. With the high rates of irregular migration to Europe, research on the mental health of IMs should be prioritised, in particular to address the identified knowledge gaps.
2. An opportunity for future research to expand upon existing knowledge would be to investigate potential social determinants of mental health outcomes in the IM population. Potential determinants may include demographic factors, reasons for migration and exposure to trauma and discrimination.
3. Methodological flaws were identified in all included studies. It is suggested that future research takes measures to address these, in order to increase the external validity of results. Suggested measures include: those to increase the representativeness samples, including less reliance on non-probability sampling, the recruitment of larger sample sizes, the use of measures validated for this study population and conducting of assessments in participant’s native language, with the use of interpreters if required.
4. Greater standardisation of research methods across this field, including the definition of IMs and the methods of measuring mental health outcomes, would allow between study comparisons, and pooling of data, in future systematic reviews.
5. Some included studies identified the presence of barriers to IMs accessing mental health care in host countries. It was found that IMs are less likely to present to health services than documented migrants, and when they do, they often do not report mental health difficulties unless asked specifically. Health services should be developed in order to be culturally-sensitive, including cultural competence training for health care professionals, in order to enhance engagement of IMs. In particular, when health care professionals are consulting with IMs they should actively and specifically ask about mental health difficulties. With the identified high rates of mental health difficulties among the IM population, policy-makers should consider prioritising access to mental health care for IMs.

## Supplementary Information

Below is the link to the electronic supplementary material.

Supplementary file1 (XLSX 31 kb)

Supplementary file2 (DOCX 22 kb)

Supplementary file3 (DOCX 20 kb)

Supplementary file4 (DOCX 13 kb)

Supplementary file5 (DOCX 30 kb)

Supplementary file6 (DOCX 17 kb)

Supplementary file7 (DOCX 15 kb)

Supplementary file8 (DOCX 15 kb)

## References

[1] United Nations. International Migration 2020 Highlights. 2021a (https://www.un.org/en/desa/international-migration-2020-highlights) Accessed 7 February 2021

[2] International Organization for Migration. World Migration Report 2010. 2010 (https://publications.iom.int/system/files/pdf/wmr_2010_english.pdf). Accessed 14 February 2021

[3] European Commission. Irregular migration. (https://ec.europa.eu/home-affairs/what-we-do/networks/european_migration_network/glossary_search/irregular-migration_en). Accessed 5 December 2020

[4] Migration Data Portal**.** Irregular migration. (https://migrationdataportal.org/themes/irregular-migration). Accessed 20 March 2021.

[5] International Organization for Migration. World Migration Report 2020. 2019 (https://publications.iom.int/system/files/pdf/wmr_2020.pdf). Accessed 5 February 2021

[6] United Nations. Migration. 2021b (https://www.un.org/en/sections/issues-depth/migration/index.html) Accessed 11 February 2021

[7] World Health Organisation. Public health aspects of mental health among migrants and refugees: a review of the evidence on mental health care for refugees, asylum seekers and irregular migrants in the WHO European region. 2016. (https://apps.who.int/iris/bitstream/handle/10665/326308/9789289051651-eng.pdf?sequence=1&isAllowed=y) Accessed 13 February 2021 27809423

[8] Blackmore R, Boyle J, Fazel M, Ranasinha S, Gray JM, Fitzgerald G, Misso M, Gibson-Heim M (2019). The prevalence of mental illness in refugees and asylum seekers: A systematic review and meta-analysis. PLoS Med.

[9] Garcini LM, Murray KE, Zhou A, Klonoff EA, Myers MG, Elder JP (2016). Mental health of undocumented immigrant adults in the United States: A systematic review of methodology and findings. J Immigr Refug Stud.

[10] Chen W, Hall BJ, Ling L, Renzaho AMN (2017). Pre-migration and post-migration factors associated with mental health in humanitarian migrants in Australia and the moderation effect of post-migration stressors: findings from the first wave data of the BNLA cohort study. Lancet Psychiatry.

[11] Satinsky E, Fuhr DC, Woodward A, Sondorp E, Robert B (2019). Mental health care utilisation and access among refugees and asylum seekers in Europe: a systematic review. Health Policy.

[12] Straßmayr C, Matanov A, Priebe S, Barros H, Canavan R, Diaz-Olalla JM, Gabor E, Gaddini A, Gracen T, Holcnerova P, Kluge U, Welbel M, Nicaise P, Shene AH, Soares J, Katschnig H (2012). Mental health care for irregular migrants in Europe: Barriers and how they are overcome. BMC Public Health.

[13] PRISMA. PRISMA Statement. 2015 (http://www.prisma-statement.org/PRISMAStatement/). Accessed 26 December 2020

[14] Garces-Mascarenas B, Penninx R (2016). Integration processes and policies in Europe contexts levels and actors.

[15] Moskalewicz A, Oremus M (2020). No clear choice between Newcastle-Ottawa Scale and Appriasal Tool for Cross-Sectional Studies to assess methodological quality in cross-sectional studies of health-related quality of life and breast cancer. J Clin Epidemiol.

[16] Naimo M, Massagli A, Degortes D, Favaro A, Campagnola N, Vidotto G (2006). The psychometric and psychosocial dimension of Albanian immigration: Data from a preliminary study. G Ital Med Lav Ergon.

[17] Heeren M, Wittmann L, Ehlert U, Schnyder U, Maier T, Muller J (2014). Psychopathology and resident status - comparing asylum seekers, refugees, illegal migrants, labor migrants, and residents. Compr Psychiatry.

[18] Teunissen E, Van Den Bosch L, Van Bavel E, Mareeuw FD, Van Den Muijsenbergh M, Van Weel-Baumgarten E, Van Weel C (2014). Mental health problems in undocumented and documented migrants: a survey study. Fam Pract.

[19] Myhrvold T, Smastuen MC (2017). The mental healthcare needs of undocumented migrants: an exploratory analysis of psychological distress and living conditions among undocumented migrants in Norway. J Clin Nurs.

[20] Andersson LMC, Hjern A, Ascher H (2018). Undocumented migrants in Sweden: mental health and associated factors. BMC Public Health.

[21] Angeletti S, Ceccarell G, Bazzardi R, Fogolari M, Vita S, Antonelli F, De Florio L, Khazrai YM, De Noia V, Lopalco B, Abacioglu H, Ciccozzi M, Berardi J, Scapaticci L, Mazzoccato V, Seguiti C, Schiavetti F, Giubilo F, Andrei N, Fricchione S, Di Renzo V, Fraschetti F, Liutakova D, Lapata S (2020). Migrants rescued on the Mediterranean Sea route: nutritional, psychological status and infectious disease control. J Infect Dev Ctries.

[22] Schoevers MA, Can Den Muijsenbergh ME, Largo-Janssen AL (2009). Self-rated health and health problems of undocumented immigrant women in the Netherlands: a descriptive study. J Public Health Policy.

[23] Sousa E, Agudelo-Suarez A, Benvides FG, Schenker M, Garcia AM, Benach J, Delclos C, Lopez-Jacob MJ, Ruiz-Frutos C, Ronda-Perez E, Porthe V (2010). Immigration, work and health in spain: the influence of legal status and employment contract on reported health indicators. Int J Public Health.

[24] Stoll CRT, Izadi S, Fowler S, Green P, Suls J, Colditz GA (2019). The value of a second reviewer for the study selection in systematic reviews. Res Synth Methods..

[25] Kessler RC, Aguilar-Gaxiola S, Alonso J, Chatterji S, Lee S, Ormel J, Bedirhan Ustun T, Wang PW (2009). Wang PW The global burden of mental disorders: an update from the WHO World Mental Health Surveys. EPS.

[26] Koenen KC, Ratanatharathorn A, Ng L, McLaughlin KA, Bromet EJ, Stein DJ, Karam EG, Meron Ruscio A, Benjet C, Scott K, Atwolli L, Petukhova M, Lim CCW, Ciutan M, De Girolamo G, Degenhardt L, Gureje O, Haro JM, Huang Y, Kawakami N, Lee S, Navarro-Mateu F, Pennell BE, Piazza M, Sampson N, Ten Have M, Torres Y, Viana MC, Williams D, Xavier M, Kessler R (2017). Postraumatic stress disorder in the World Mental Health Surveys. Psychol Med.

