# Supplementary material for: The Mental Health of Adult Irregular Migrants to Europe: A Systematic Review
Source: J Immigr Minor Health. 2022 Jul 15;25(2):427–35. doi: 10.1007/s10903-022-01379-9 (PMC9988753; doi:10.1007/s10903-022-01379-9)
Supplement: Supplementary file 2 — Supplementary file2 (DOCX 22 kb) [file 10903_2022_1379_MOESM2_ESM.docx]

**Appendix 1**

**Systematic review protocol**

**Title**

The mental health of adult irregular migrants to Europe: A systematic review

**Questions**

1. What are the mental health outcomes in adult irregular migrants (IMs) to Europe?
2. How do adult IMs to Europe manage mental health difficulties?

*Mental health outcomes are defined as: the nature and prevalence of mental health difficulties. Mental health difficulties are defined as: psychological symptoms, diagnosis with a mental disorder, or poor overall mental health.*

**PICOS**

**Population**

Adult IMs to Europe

**Intervention**

-

**Context**

European countries

**Outcome**

Mental health

**Study design**

Quantitative studies

**Rationale**

Irregular migration to Europe is high; the number of IMs arriving to Europe by sea in 2018 was 144,166 (4). IMs are exposed to risk factors for mental health difficulties, and face barriers to accessing mental healthcare in their host countries. Previous research has found that some migrant groups may experience higher rates of certain mental health difficulties, in particular PTSD, compared with non-migrants. No previous systematic reviews on the mental health of adult IMs to Europe were identified.

**Selection criteria**

**Inclusion criteria**

**Population**

- Adult (≥18 years old) IMs to Europe

*Irregular migration was defined from the perspective of host countries as “entry, stay or work in a country without the necessary authorisation or documents required under immigration regulations” (3).*

**Outcome**

- Nature and prevalence of mental health difficulties

**Context**

- European countries

**Study design**

- Quantitative studies
- Published on 1^st^ January 1990 or later

*This time frame was chosen because the collapse of the Iron Curtain and the end of the Cold War occurred around 1990, which led to an increase in migration in Europe (14).*

**Exclusion criteria**

**Population**

- Studies with samples that were recruited through mental health services
- Studies in which data for adult IMs was not separated from that for:
  - Children and adolescents (<18 years old)
  - Other migrant groups
  - Detained migrants

**Outcome**

- Studies investigating substance use, but not other mental health outcomes
- Studies that do not report the nature and prevalence of mental health outcomes

**Context**

- Non-European countries, including Israel

**Study design**

- Studies with only qualitative data
- Intervention studies

**Language**

- Unable to obtain an English language version

**Types of literature**

- Non-peer reviewed literature, including grey literature, commentary articles, reviews, editorials and letters
- Where a single data set is reported in multiple articles, only the article which best meets the selection criteria will be included
- Unable to access full-text through the University of Glasgow institutional login or library services

**Search strategy**

**Population**

**Keywords** (OVID)

migrant* OR immigra* OR emigrant* OR “displaced person*” OR transient* OR alien* OR migration OR “displaced people” OR “displaced population*” OR displaced ADJ2 (person OR people OR population)

AND

undocumented OR illegal OR unlawful OR unauthori#ed OR irregular OR “immigration status” OR “documentation status” OR paperless OR (refused ADJ asylum) OR insecur* OR “sans papiers” OR “sin papeles”

**Subject headings**

“undocumented immigrants” (MEDLINE)

“undocumented immigrant” (EMBASE)

“illegal immigrants” (CINAHL)

**Intervention**

-

**Control**

-

**Outcome**

**Keywords** (OVID)

mental ADJ/N2 (health OR disorder* OR illness* OR hygiene)) OR psychiatr* OR psycholog* OR psychopathology OR “self#harm” OR suicid* OR affective OR mood OR depress* OR anxi* OR neurosis OR neurotic OR (trauma ADJ4 (symptom* OR disorder*)) OR “post traumatic stress*” OR PTSD OR PTSS OR psychosis OR psychotic OR schizophreni* OR schizoaffective OR bipolar OR “personality disorder” OR dissociation OR somatoform OR (sleep ADJ3 disorder*) OR “eating disorder”

**Subject headings**

“mental health”, “mental disorders” (MEDLINE and CINAHL)

“mental health”, “mental disease” (EMBASE)

**Context**

-

**Study design**

-

**Databases/ Search engines**

- MEDLINE (OVID)
- EMBASE (OVID)
- CINAHL (EBSCO)
- PsychINFO (EBSCO)

**Critical appraisal**

Appraisal tool for Cross-Sectional Studies (AXIS)

*All of the studies included in this systematic review were of a cross-sectional design. AXIS is* *the only formal tool for the critical appraisal of cross-sectional studies.*

Grading system: high quality for >80% score, moderate for 50-79% and low for <50%

*There is no grading system for AXIS, and therefore this system was developed for this systematic review.*

**Data extraction**

Data was extracted and entered into an Excel spreadsheet with the following headings:

Study (Author(s) and year)

Year(s) conducted

Organisation conducting the research

Funding source

Aim

Study design

Definition of IM

Host country/ region

Sampling method

Sample size (including breakdown of different groups is applicable)

Method of measuring mental health outcomes

Location of assessment

Role of assessors

Assessment in native language

Statistical methods

Country/ region of origin

Age

Sex

Religion

Relationship status

Children (if yes, separated?)

Trauma exposure (pre-migration)

Mental health difficulties (pre-migration)

Reasons for migration

Separation from relatives

Relatives in host country

Duration of displacement

Indicators of socioeconomic status (post-migration)

Physical health difficulties (post-migration)

Trauma exposure (post-migration)

Prevalence of depression

Prevalence of anxiety

Prevalence of PTSD

Prevalence of other mental health difficulties

Within sample comparisons of mental health outcomes

Examples of how irregular migrants have managed mental health difficulties

Prevalence of adoption of strategies to manage mental health difficulties

Prescription of psychotropic medications

Other results of interest

Overall conclusions
