# Supplementary material for: The Mental Health of Adult Irregular Migrants to Europe: A Systematic Review
Source: J Immigr Minor Health. 2022 Jul 15;25(2):427–35. doi: 10.1007/s10903-022-01379-9 (PMC9988753; doi:10.1007/s10903-022-01379-9)
Supplement: Supplementary file 4 — Supplementary file4 (DOCX 13 kb) [file 10903_2022_1379_MOESM4_ESM.docx]

**Appendix 3**

**Additional characteristics of included studies**

| **Study** | **Sample composition** |
| --- | --- |
| **Naimo et al. (2006)** | Migrants who entered Italy legally; migrants who entered Italy illegally |
| **Schoevers et al. (2009)** | Undocumented migrants |
| **Sousa et al. (2010)** | Spanish born workers (permanent contract; temporary contract; no contract); foreign-born workers (documented; temporary contract; documented; no contract; undocumented) |
| **Heeren et al. (2014)** | Residents; labour migrants; refugees holding permanent protection visas; asylum-seekers; illegal migrants |
| **Teunissen et al. (2014)** | Documented migrants; undocumented migrants |
| **Myhrvold and Smastuen (2017)** | Undocumented migrants |
| **Andersson et al. (2018)** | Undocumented migrants |
| **Angeletti et al. (2020)** | Migrants rescued in the Mediterranean Sea after attempting to cross by boat from Libya |
