# Supplementary material for: The Mental Health of Adult Irregular Migrants to Europe: A Systematic Review
Source: J Immigr Minor Health. 2022 Jul 15;25(2):427–35. doi: 10.1007/s10903-022-01379-9 (PMC9988753; doi:10.1007/s10903-022-01379-9)
Supplement: Supplementary file 5 — Supplementary file5 (DOCX 30 kb) [file 10903_2022_1379_MOESM5_ESM.docx]

**Appendix 4**

**Appraisal of cross-sectional studies (AXIS) of included studies**

| Study | | *Naimo et al. (2006)* | *Schoevers et al. (2009)* | *Sousa et al. (2010)* | *Heeren et al. (2014)* | *Teunissen et al. (2014)* | *Myhrvold and Smastuen (2017)* | *Anderssonet al. (2018)* | *Angeletti et al. (2020)* |
| --- | --- | --- | --- | --- | --- | --- | --- | --- | --- |
| *Introduction* | | | | | | | | | |
| 1 | Were the aims/ objectives of the study clear? | Yes | Yes | Yes | Yes | Yes | Yes | Yes | No |
| *Methods* | | | | | | | | | |
| 2 | Was the study design appropriate for the stated aim(s)? | Yes | Yes | Yes | Yes | Yes | Yes | Yes | Don’t know |
| 3 | Was the sample size justified? | No | No | No | No | No | No | No | No |
| 4 | Was the target/ reference population clearly defined? (Is it clear who the research was about?) | Yes | Yes | Yes | Yes | Yes | Yes | Yes | No |
| 5 | Was the sample frame taken from an appropriate population base so that it closely represented the target/ reference population under investigation? | No | No | No | No | No | No | No | No |
| 6 | Was the selection process likely to select subjects/ participants that were representative of the target/ reference population under investigation? | No | No | No | No | No | No | No | No |
| 7 | Were measures undertaken to address and categorise non-responders? | No | No | No | No | Not applicable | No | No | Not applicable |
| 8 | Were the risk factor and outcome variables measured appropriate to the aims of the study? | Yes | Yes | Yes | Yes | Yes | Yes | Yes | Don’t know |
| 9 | Were the risk factor and outcome variables measured correctly using instruments/ measurements that had been trialled, piloted or published previously? | Yes | No | Yes | Yes | Yes | Yes | Yes | Yes |
| 10 | Is it clear what was used to determine statistical significance and/ or precision estimates? (eg. p-values, confidence intervals) | Yes | Yes | Yes | Yes | Yes | Yes | Yes | No |
| 11 | Were the methods (including statistical methods) sufficiently described to enable them to be repeated? | Yes | Yes | Yes | Yes | Yes | Yes | Yes | No |
|  | |  |  |  |  |  |  |  |  |
| Study | | ***Naimo et al. (2006)*** | ***Schoevers et al. (2009)*** | ***Sousa et al. (2010)*** | ***Heeren et al. (2014)*** | ***Teunissen et al. (2014)*** | ***Myhrvold and Smastuen (2017)*** | ***Anderssonet al. (2018)*** | ***Angeletti et al. (2020)*** |
| *Results* | | | | | | | | | |
| 12 | Were the basic data adequately described? | Yes | Yes | Yes | Yes | Yes | Yes | Yes | Yes |
| 13 | Does the response rate raise concerns about non-response bias? | Don’t know | Don’t know | No | No | No | No | Yes | No |
| 14 | If appropriate, was information about non-responders described? | No | No | No | No | Not applicable | No | No | Not applicable |
| 15 | Were the results internally consistent | Yes | Yes | Don’t know | Yes | Yes | Yes | No | Yes |
| 16 | Were the results presented for all the analyses described in the methods? | Yes | Yes | Yes | Yes | Yes | Yes | Yes | Yes |
| *Discussion* | | | | | | | | | |
| 17 | Were the authors’ discussions and conclusions justified by the results? | Yes | Yes | Yes | Yes | Yes | Yes | Yes | Yes |
| 18 | Were the limitations of the study discussed? | No | Yes | Yes | Yes | Yes | Yes | Yes | No |
| *Other* | | | | | | | | | |
| 19 | Were there any funding sources of conflicts of interest that may affect the author’s interpretation of the results? | Don’t know | Don’t know | No | Don’t know | No | No | No | No |
| 20 | Was ethical approval or consent of participants attained? | Don’t know | Yes | Don’t know | Yes | Yes | Yes | Yes | Don’t know |
| *Overall score* | | 11 | 12 | 12 | 13 | 17 | 14 | 13 | 8 |
| *Grade* | | Moderate | Moderate | Moderate | Moderate | High | Moderate | Moderate | Low |
