# Supplementary material for: The Mental Health of Adult Irregular Migrants to Europe: A Systematic Review
Source: J Immigr Minor Health. 2022 Jul 15;25(2):427–35. doi: 10.1007/s10903-022-01379-9 (PMC9988753; doi:10.1007/s10903-022-01379-9)
Supplement: Supplementary file 6 — Supplementary file6 (DOCX 17 kb) [file 10903_2022_1379_MOESM6_ESM.docx]

**Appendix 5**

**Additional characteristics of irregular migrant participants in included studies 1.0**

| **Study** | **Female (%)** | **Age** | **Country/ region of origin** | **Relationship status** | **Children** | **Education** |
| --- | --- | --- | --- | --- | --- | --- |
| **Naimo et al. (2006)** | 30.77 | Mean 35.1 years (SD 12.0; range 18-64) | Albania | Single 35.4%; married 58.5%; other 4% | - | Mean duration 12.6 years (SD 3.15) |
| **Schoevers et al. (2009)** | 100 | Mean 36.4 years (SD 14.7) | Eastern Europe/ former USSR 30%; Sub-Saharan Africa 21%; Turkey/ Middle East/ Northern Africa 12%; China/ Mongolia 12%; Afghanistan/ Iran 11%; Middle and South America/ Philippines 8%; Surinam 6% | No partner 47%; partner 53% | Yes 73%; no 27% | Able to read and write 81%; difficulties with reading and writing 8%; illiterate 11% |
| **Sousa et al. (2010)** | 43.4 | <30 years 65.5%; 30-39 years 34.5% | Morocco; Ecuador; Romania; Colombia | - | - | Highest level: primary 32.4%; secondary 52.3%; university 14.8% |
| **Heeren et al. (2014)** | 47.6 | Mean 37.8 years (SD 10.2) | Asia 14.3%; Africa 38.1%; South America 47.6% | Married 38.1% | Mean number of children 0.95 (SD 1.5) | Mean duration 10.4 years (SD 5.5) |
| **Teunissen et al. (2014)** | 36.9 | 18-30 years 29.5%; 31-50 years 51.7%; >50 years 18.8% | Sub-Saharan Africa 24.3%; Turkey/ Middle East/ Northern Africa 30.2%; Afghanistan/ Iran/ Iraq/ Pakistan 6.5%; Eastern Europe (non-EU)/ former Union of Soviet Socialist Republics/ former Yugoslavia 3.7%; Middle and South America 14.5%; Surinam 0.6%; Asia 4%; Unknown 16.3% | - | - | - |
| **Myhrvold and Smastuen (2017)** | 43 | 18-29 years 32%; 30-39 years 43%; >40 years 25% | Mongolia 20%; Ethiopia 14.5%; Afghanistan 13.3%; Iran 11.1%; Somalia 3.3%; 18 more countries 37.8% | Single 43%; married/ steady partner 37%; divorced, separated or widowed 20% | Children 48%; no children 52% | No formal education 8%. Highest level: primary 10%; lower secondary 26%; upper secondary 26%; degree 29% |
| **Andersson et al. (2018)** | 45.5 | 18-24 years 22.7%; 25-39 years 52.3%; >40 years 23.9% | Afghanistan 28.4%; Middle East 18.2%; Former Yugoslav republics 15.9%, Former Soviet republics in Asia and Europe 14.8%; West and Central Africa 4.5%; East Africa 6.8%; North Africa 3.4%; Other 8% | Single/widowed 54%; married/cohabiting/steady relationship 46% | Child in Sweden 47.3%; child in another country 5.3%; no children 43.2% | Highest level: primary 40.9%; secondary 21.6%; college/ university 37.5% |
| **Angeletti et al. (2020)** | 8 | Median 24 years | Eritrea | Unmarried 67% | - | Median duration 10 years (Range 0-16) |

Abbreviations: standard deviation (SD); European Union (EU)
