# Supplementary material for: The Mental Health of Adult Irregular Migrants to Europe: A Systematic Review
Source: J Immigr Minor Health. 2022 Jul 15;25(2):427–35. doi: 10.1007/s10903-022-01379-9 (PMC9988753; doi:10.1007/s10903-022-01379-9)
Supplement: Supplementary file 7 — Supplementary file7 (DOCX 15 kb) [file 10903_2022_1379_MOESM7_ESM.docx]

**Appendix 6**

**Additional characteristics of irregular migrant participants in included studies 1.1**

| **Study** | **Trauma exposure** | **Reasons for migration** | **Duration of displacement** | **Indicators of socioeconomic status (post-migration)** | **Physical health difficulties** |
| --- | --- | --- | --- | --- | --- |
| **Naimo et al. (2006)** | In Albania: 56.41%. During the journey: violence 3%; psychological humiliation and ill-treatment 10%; witnessed violence against a fellow passenger by a pilot 15%; witnessed the wounding or death of a fellow passenger 10% | Economic and employment 82%; political and religious 13%; “in order to have a better life” 15%; “to provide a future for the children” 15% | >1 year 100% | Employed 38% | - |
| **Schoevers et al. (2009)** | Physical violence 43%; sexual violence 28%; female genital mutilation 3%; involuntary prostitution 5% | Political 58%; non-political 42%; economic 14%; personal 29% | Mean 7 years (SD 3.7) | Employed 20%; student 1%; unemployed 79%. Semi-permanent residence 24%; temporary residence 69%; homeless 7% | Poor self-rated health 65%. Participants reported having an average of 11.1 health problems |
| **Sousa et al. (2010)** | - | - | ≤3 years 61.8%; >3 years 38.2% | Monthly income: ≤€600 30.8%; €601-1200 63.2%; €1201-1800 26%; >€1800 0% | Males: poor self-rated health 28.4%. Females: poor self-rated health 50.6% |
| **Heeren et al. (2014)** | Mean lifetime traumatic event exposure types 2.6 (SD 4.2) | - | Mean 5 years (SD 3.3) | Employed 52.4%. High post-migration resources 33.3% | - |
| **Teunissen et al. (2014)** | - | - | - | - | - |
| **Myhrvold and Smastuen (2017)** | Incidents of sexual abuse, violence and harassment: never 45%; would not answer 13.5%; at least one 41.5% | War 28%; persecution 25.5%; economic insecurity 22%; other 24.5% | Median 4 years (range 1 month-31 years) | Financial dependence: yes 61%; no 39%. Debt: yes 38%; no 51%; would not answer 11%. Homeless: yes 25%; no 75%. Employment status: never worked in Norway 36%; work affiliation 64%. Go to bed hungry at least once a month: yes 60%; no 40% | - |
| **Andersson et al. (2018)** | - | Belonged to a harassed minority 51.1%; political 46.6%; war 42%; religious 31.8%; social 28.4%; honour-related 27.2%; economic 23.9%; disease 11.3%; none of the above 5.7%; sexual orientation 2.2% | <12 months 27.2%; 12–24 months 40.9%; >2 years 31.8% | Homeless or temporary accommodation 30.7%; fairly stable accommodation 69.3%. Has often gone hungry 56.8% | Longstanding physical illness 55.8% |
| **Angeletti et al. (2020)** | They all reported that they had been prisoners in Libya, where they were neglected, maltreated and physically abused. 7% were hospitalised on arrival in Italy for trauma caused by repeated rape | - | Range 11-21 months | - | 15.8% were hospitalised on arrival in Italy. 50% were underweight (BMI < 18.5) |

Abbreviations: standard deviation (SD); tuberculosis (TB); body mass index (BMI); hepatitis (hep)
