# Supplementary material for: The Mental Health of Adult Irregular Migrants to Europe: A Systematic Review
Source: J Immigr Minor Health. 2022 Jul 15;25(2):427–35. doi: 10.1007/s10903-022-01379-9 (PMC9988753; doi:10.1007/s10903-022-01379-9)
Supplement: Supplementary file 8 — Supplementary file8 (DOCX 15 kb) [file 10903_2022_1379_MOESM8_ESM.docx]

**Appendix 7**

**Additional mental health outcomes for irregular migrant participants in included studies**

| **Study** | **Prevalence of other mental health difficulties** | **Within sample comparisons** |
| --- | --- | --- |
| **Naimo et al. (2006)** | - | Females experienced a greater frequency of MDD (*p*=0.01) |
| **Schoevers et al. (2009)** | 31 of participants had been pregnant whilst they were an IM, 10% of these had experienced perinatal depression and/or PTSS | - |
| **Sousa et al. (2010)** | 51.3% of males and 50.6% of females had poor mental health | Undocumented males who had lived in Spain for ≤3 years had worse mental health than Spanish-born permanently contracted (aOR 2.26, CI 95% 1.15–4.42) |
| **Heeren et al. (2014)** | - | Asylum-seekers had higher rates of depression compared with IMs (*p*<0001); IMs had higher rates than labour migrants (*p*<0.05) and residents (*p*<0.01). IMs had higher rates of anxiety than labour migrants and residents (*p*<0.01). Asylum-seekers had higher rates of PTSD than IMs (*p*<0.001) |
| **Teunissen et al. (2014)** | Sleeping disorder 1.5%; addiction 3.1%; psychosis 1.8%. At least one P-code 20.6%. At least one tag 11.1% | Undocumented migrants contacted their GP less often than documented migrants (3.1 times a year versus 4.9). 44% of documented migrants had at least one P-code (*p*=0.00), 8.3% at least one tag, 5.6% a sleeping disorder (*p*=0.012), 9.3% an addiction (*p*=0.002); and 5.1% psychosis (*p*=0.047), compared with undocumented migrants |
| **Myhrvold and Smastuen (2017)** | 87% had emotional distress in need of diagnostic evaluation and mental health care | ‘Leaving home country because of war or persecution’ (*p*<0.001), ‘having someone ﬁnancially dependent on you’ (*p*<0.04), ‘being homeless’ (*p*<0.04), ‘hunger’ (*p*<0.01) and ‘having experienced at least one incident of sexual or other harassment’ (*p*<0.03) were associated with higher levels of psychological distress. ‘Higher level of education’ was associated with a reduction (*p*<0.01) |
| **Andersson et al. (2018)** | - | Being aged 40 years or more was associated with higher depression scores (*p*<0.05), and the 25-39 years age group had higher anxiety scores than the 18-24 years group (*p*<0.05). Insecure housing was a risk factor for depression and anxiety (*p*<0.05) |
| **Angeletti et al. (2020)** | 100% screened positive for symptoms of common mental disorders, which require further assessment (Cronbach‘s α 0.95) | - |

Abbreviations: irregular migrant (IM); general practitioner (GP); post-traumatic stress disorder (PTSD); post-traumatic stress syndrome (PTSS); major depressive disorder (MDD); Psychological International Classification of Primary Care code (P-code); mental health difficulties documented in general practice records, but not coded (tags); adjusted odds ratio (aOR); confidence interval (CI)
